# Chronic Pain after Bone Fracture: Current Insights into Molecular Mechanisms and Therapeutic Strategies

**DOI:** 10.3390/brainsci12081056

**Published:** 2022-08-09

**Authors:** Yuying Zhao, Haoyue Zhang, Nan Li, Jing Li, Linlin Zhang

**Affiliations:** 1 Department of Anesthesiology, Tianjin Medical University General Hospital, Tianjin 300052, China; 2 Tianjin Research Institute of Anesthesiology, Tianjin 300052, China

**Keywords:** caspases, chronic fracture pain, neuroinflammation, spinal dorsal horn, STING, synaptic plasticity

## Abstract

Bone fracture following traumatic injury or due to osteoporosis is characterized by severe pain and motor impairment and is a major cause of global mortality and disability. Fracture pain often originates from mechanical distortion of somatosensory nerve terminals innervating bones and muscles and is maintained by central sensitization. Chronic fracture pain (CFP) after orthopedic repairs is considered one of the most critical contributors to interference with the physical rehabilitation and musculoskeletal functional recovery. Analgesics available for CFP in clinics not only have poor curative potency but also have considerable side effects; therefore, it is important to further explore the pathogenesis of CFP and identify safe and effective therapies. The typical physiopathological characteristics of CFP are a neuroinflammatory response and excitatory synaptic plasticity, but the specific molecular mechanisms involved remain poorly elucidated. Recent progress has deepened our understanding of the emerging properties of chemokine production, proinflammatory mediator secretion, caspase activation, neurotransmitter release, and neuron-glia interaction in initiating and sustaining synaptogenesis, synaptic strength, and signal transduction in central pain sensitization, indicating the possibility of targeting neuroinflammation to prevent and treat CFP. This review summarizes current literature on the excitatory synaptic plasticity, microgliosis, and microglial activation-associated signaling molecules and discusses the unconventional modulation of caspases and stimulator of interferon genes (STING) in the pathophysiology of CFP. We also review the mechanisms of action of analgesics in the clinic and their side effects as well as promising therapeutic candidates (e.g., specialized pro-resolving mediators, a caspase-6 inhibitor, and a STING agonist) for pain relief by the attenuation of neuroinflammation with the aim of better managing patients undergoing CFP in the clinical setting.

## 1. Introduction

Bone fractures, resulting from orthopedic trauma or osteoporosis, contribute to increasing rates of morbidity, disability, mortality, and medical expenditures worldwide. According to statistics, the annual number of patients who experience a traumatic fracture is over 4,400,000 in China [1], and about 9,000,000 people per year experience an osteoporotic fracture worldwide [2].

Chronic pain is considered to be pain that persists beyond the normal healing time, usually referring to pain lasting >3 months. Chronic fracture pain is one of the common clinical chronic pains, which is characterized by severe pain during exercise, mechanical allodynia, and cold hyperalgesia [3]. Notably, the incidence rate of chronic pain is 61.7% after ankle and knee fractures [4] and 55.1% after tibial fractures [5]. Patients with CFP not only have poor musculoskeletal functional recovery but also experience depression, anxiety, cognitive impairments, and complex regional pain syndrome (CRPS), which have been identified as related to worsening pain perception and a serious threat to quality of life [6,7].

Fracture pain often originates from mechanical distortion of somatosensory nerve terminals innervating bones and muscles. Then, the initial fracture pain signal is emitted by activated mechanosensors expressed by nerve fibers densely innervating the periosteum [8]. Fracture patients are known to experience mechanical allodynia in the affected limb [9]. Mechanical allodynia as well as thermal hyperalgesia have also been described in a mouse fracture pain model [10,11]. CFP appears to be involved in the body’s abnormal response to tissue damage. The typical physiopathological characteristics include a neuroinflammatory response and excitatory synaptic plasticity, involving chemokine production, proinflammatory mediator secretion, caspase activation, neurotransmitter release, and neuron-glia interaction. These factors play essential roles in initiating and sustaining synaptogenesis, synaptic strength, and signal transduction during central pain sensitization. The molecular mechanisms of CFP remain not well elucidated, which is a hot topic in the pain research field.

Currently, non-steroidal anti-inflammatory drugs (NSAIDs) and opioids remain the most commonly used analgesics, but their serious side effects are also prominent problems that endanger the physical and mental health of patients. Therefore, in-depth research on the mechanism that mediates the production of CFP in order to develop more targeted drugs is a current and even future research focus.

This review focuses on the molecular pathways that are clearly involved in the regulation of CFP, the characteristics, and side effects of commonly used clinical analgesics and the research prospects of some potential molecular targets, aiming to provide new ideas for high-efficiency CFP and the development of efficient CFP-targeted drugs.

## 2. CFP Preclinical Animal Model

The development and treatment of orthopedic illnesses have long been the subject of effective preclinical animal research, which is a crucial link in this process. Orthopedic disease models have undergone constant improvement throughout time. From the early closed femoral fracture model to the more complete closed fracture model and intramedullary nail placement [12] as well as open fracture models [13], bone-related infection models [14], atrophic nonunion models [15], etc., a variety of models have been developed and applied to orthopedic disease-related research [16]. In this article, we focus on describing the use of three fracture models to study the pathogenesis of CFP.

Fracture models are typically performed using rodent long bones (usually the tibia or femur). The skin of the leg is shaved and sterilized after the experimental animal is anesthetized. To reveal the bone, the surgical site’s skin is first longitudinally sliced, and the muscle is then dissected. An intramedullary nail is built into the bone marrow to stabilize the mouse bone. The size of the intramedullary nail is generally selected according to the thickness of the test animal’s bones (generally 1–7 mm for rats and 0.1–4 mm for mice). After the tibia is exposed, the mouse tibia is manually cut after the above-mentioned steps and then sutured to create an open fracture model [13]. However, only the tibial plateau is exposed, and after the above treatment, the tibia is sutured first, then it is broken with a weight or three-point bending forceps to obtain a closed fracture model, with no obvious wound on the skin of the broken leg [12].

On the basis of the fracture model, the osteoporotic fracture model was developed to study another common fracture in clinical practice, the osteoporotic fracture [17]. This model is mostly made in female mice (because osteoporosis is more common in postmenopausal women). After surgical removal of the ovaries, the mice are bred for 4 weeks and then treated with fractures to obtain the target model.

These are the three most commonly used fracture models in the research process. However, the growth rate of rats is almost tens or even hundreds of times greater than that of humans, according to research. Therefore, preclinical studies of chronic pain (>3 months) typically only need to observe changes in rodents within 1 month (usually 21/28 days) after surgery. Based on this, the study of CFP can effectively clarify its working principle and also promote the exploration of high-efficiency analgesics.

## 3. Molecules Involved in CFP and Their Mechanisms of Action

### 3.1. AMPA Receptor Cascades

AMPA (α-amino-3-hydroxy-5-methyl-4-isoxazole propionic acid) receptors belong to the group of ionotropic glutamate receptors, which are concentrated in the excitatory postsynaptic membrane in and out of synapses in a constitutive and activity-dependent manner [7]. AMPA receptors are essentially tetrameric ion channels assembled from four subunits: GluA1, GluA2, GluA3, and GluA4 [18]. Each subunit has a different role in the mammalian central nervous system. 

After in-depth research, it was found that AMPA receptors, especially AMPA receptors containing GluA1, are the core of the regulation of excitatory synaptic transmission in the central nervous system (CNS) and are an indispensable part of the formation of chronic pain [19,20,21].

#### 3.1.1. Caspase-3

Caspase-3 has been called the executioner caspase in apoptosis. Active Caspase-3 is present in postsynaptic structures and plays an important role in regulating synaptic plasticity (structural remodeling and long-term functional changes) [22]. Leucine-rich repeat transmembrane protein 1 (LRRTM1), a synaptic cell adhesion molecule located at the postsynaptic membrane, is one of the most critical candidates for inducing excitatory synaptogenesis and maintaining normal synaptic function [23]. The study by Wu F et al. demonstrated that the caspase-3 inhibitor Z-DEVD-FMK or caspase-3 siRNA reduced chronic pain after nerve injury [24]. In our study [21], CFP was inhibited after using Z-DEVD-FMK or knocking out LRRTM1, reaffirming that caspase-3 activation and LRRTM1 overexpression promote the development of CFP in mice. However, how caspase-3 specifically affects the continuous activation of CFP and its underlying epigenetic mechanism has not yet been elucidated; thus, this requires further study.

#### 3.1.2. Caspase-6

Caspase-6 is specifically distributed in the axonal terminals of superficial C fibers in the dorsal horn of the spinal cord and is co-expressed with calcitonin gene-related peptide (CGRP). After injury, caspase-6, located in peptidergic primary afferent neurons, can be transported to the central terminal of the spinal cord, where it participates in the maintenance of pathological pain and the functional enhancement of excitatory synapses [25]. Caspase-6 activation may be a major feature of neuroinflammation and neuron-microglia interaction and a key driver of synaptic plasticity and central sensitization [25,26]. Intrathecal injection of recombinant caspase-6 was sufficient to induce symptoms such as mechanical allodynia, and pretreatment with the microglia inhibitor minocycline reversed these symptoms, confirming the effect of caspase-6 on microglia [27]. In vitro culture of primary microglia and administration of recombinant caspase-6 resulted in a massive release of TNF-α [28], whereas p38 inhibitor treatment of microglia inhibited caspase-6-induced TNF-α release [29]. Furthermore, intrathecal injection of the caspase-6 inhibitor Z-VEID-FMK or a caspase-6-neutralizing antibody also alleviated CFP by inhibiting AMPA receptor transport and spinal dorsal horn dendritic spine remodeling [19]. All of the above findings indicate that caspase-6 plays an irreplaceable role in the development of CFP. However, how caspase-6 functions in microglia after it is released from axon terminals and what the underlying gene expression mechanism is remain to be further explored.

### 3.2. NMDA Receptor Cascades

Excitatory glutamatergic NMDA (N-methyl-D-aspartate) receptors are key regulators of excitability and plasticity in the superficial spinal dorsal horn. Functional NMDA receptors are obligate heteromers that are usually composed of two NR1 and two NR2 subunits [30]. NR2 consists of four subunits, and selective activation of NR2B-containing NMDA receptors triggers excitotoxicity and synaptic plasticity [31,32]. Accumulation of NR2B at Try1472 is essential for NMDA receptor hyperexcitation, strength of glutamatergic synapses, and central sensitization in pain-related syndromes [33,34]. 

Divalent metal transporter 1 (DMT1) is a transmembrane ferroprotein that is responsible for the transport of the ferrous iron, and its regulation of iron overload has been shown to promote autophagy and apoptosis in osteoblasts [35]. Based on whether the 3′ untranslated region (UTR) contains the iron responsive element (IRE), DMT1 is defined as IRE(-)DMT1 or IRE(+)DMT1 [36]. Recent research shows that DMT1, especially IRE(-)DMT1, has been manifested to mediate iron overload in NMDA neurotoxicity [31,37].

Kalirin-7, the major isoform expressed in the adult rodent brain, is an important selective regulator involved in the regulation of NMDA receptor-dependent synaptic plasticity [38]. A recent series of studies have begun to reveal the exact function of kalirin-7 in controlling dendritic spine morphogenesis and its relevance to development, plasticity, and synaptic pathology not only by regulating synapses of AMPA receptors containing GluA1 transport to regulate neural excitotoxicity but also through directly interaction with NR2B, which is involved in synaptic transport [20,39]. Kalirin-7 knockdown or application of deferoxamine inhibited IRE(-)DMT1-mediated iron overload and spinal plasticity, thereby impairing CFP. The selective NR2B antagonist Ro25-6981 attenuated CFP, presumably by reducing kalirin-7 levels and IRE(-)DMT1-mediated iron overload that impedes spinal NR2B phosphorylation [31]. However, this study was performed with female mice, and the results may not be representative of male mice. Therefore, whether CFP production can still be inhibited by regulating kalirin-7 in male mice remains to be further investigated. Moreover, its deep genetic mechanism is another focus of our research.

### 3.3. Neuroinflammation

#### 3.3.1. The NF-κB Pathway

A potential mechanism mediating CFP is sustained activation of the inflammatory cascade in the immediate postoperative period. Nuclear factor kappa B (NF-κB) is a protein complex that regulates gene expression and cytokine production and is essential for cellular stress responses. After NF-κB activation, various complex cascades related to tissue healing occur in succession [40]. Proinflammatory mediators regulated through the NF-κB pathway are associated with pain [41]. For bone tissue, the inhibition of NF-κB is an effective means for inhibiting osteoclast activity and bone resorption [42]. The NF-κB pathway is activated or regulated by the production of interleukin [IL]1 receptor antagonist/IL-1ra, IL-6, IL-8, and TNF-α and its soluble receptors (sTNF-RⅠ and sTNF-RⅡ) [43]. These inflammatory factors can interact or act directly on nociceptors to mediate the production and development of CFP. Intraplantar injection of IL-1β could lead to mechanical allodynia via the activation of nociceptive small-diameter Aδ and C fibers [44]. IL-1β may promote nerve growth factor (NGF) overexpression in keratinocytes, thereby increasing chronic nociceptor sensitivity after fracture [45,46]. An increase in pro-inflammatory cytokines such as TNF-α in the brain can activate microglia, leading to the release of a variety of proinflammatory cytokines (IL-1ra, IL-6, IL-8) after activation. This positive feedback amplification mechanism of inflammation further exacerbates nerve cell damage and promotes the transformation of postoperative acute pain to chronic pain [44,47,48,49]. Receptor-Interacting Protein Kinase 3 (RIPK3) is an important promoter of induced mechanical allodynia. RIPK3 inhibition may alleviate mechanical allodynia by inhibiting the activation of the NLRP3 inflammasome and NF-κB, thereby reducing the release of IL-1β, IL-18, and TNF-α [41]. More studies have shown that after fracture, activated keratinocytes proliferate and overexpress inflammatory mediators such as IL-1β, IL-6, TNF-α, and NGF-β, which work together with inflammatory mediators present in the skin to cause persistent hyperalgesia. Treatment with the global cytokine inhibitor pentoxifylline, a TNF inhibitor (etanercept), an IL-1 receptor antagonist (anakinra), or an anti-NGF antibody (tanezumab) is effective in reducing CFP, and this effect lasts for 4 weeks [46]. Although many drugs used in animal experiments have obvious inhibitory effects on CFP, their clinical safety is still unknown, and further research is still needed in order to clinically reduce pain in patients at an early date.

#### 3.3.2. IL-6 Signaling

Interleukin 6 (IL-6), a well-known regulator of B-cell maturation and immunoglobulin production, is an important factor in the production of pain-related autoantibodies [50,51]. A study by Zhao Y et al. found that the serum inflammatory factor IL-6 level was significantly correlated with the degree of pain in patients [52]. Patients were found to have markedly elevated cutaneous IL-6 levels one month after fracture surgery, and local IL-6 levels persisted for several months following cast immobilization in a rodent fracture model, suggesting that IL-6 may be a key chronic immune modulator of pain [53,54]. IL-6 was shown to mediate mechanical allodynia and thermal hyperalgesia in mice by promoting nociceptor sensitization and central sensitization through direct sensitization of nociceptive neurons or by enhancing the expression of TRPV1 channels [51,55]. Subsequent initiation of induced mechanical hypersensitivity and hyperalgesia is dependent on BDNF/TrkB signaling [56,57,58]. In the same tibial fracture model, local injection of a small molecule IL-6 inhibitor was shown to rapidly reduce hindpaw sensitivity, and an intrathecal injection of anti-interleukin-6 neutralizing antibody also alleviated pain-related behaviors [59,60]. Therefore, we can predict that, in CFP patients, reducing the sensitivity of nociceptors by reducing IL-6 signaling may be an effective pain relief strategy. There are anti-IL-6 or anti-IL-6 receptor biological drugs, such as torolizumab and sarilumab [61]. Studies have shown that they have a limited analgesic effect, but long-term anti-IL-6 therapy leaves patients susceptible to infection [62]. Therefore, the development of safer and more efficient anti-IL-6 drugs is still the focus of future research, and determining how to effectively avoid the side effects of anti-IL-6 therapy needs more consideration.

#### 3.3.3. Chemokines 

Chemokine-dependent neuroinflammation plays a pivotal role in excitatory synaptic plasticity and central nociceptive sensitization. Chemokines are divided into C, CC, CXC, and CX3C subfamilies based on the conserved cysteine motifs they contain. Different chemokine subfamilies are involved in mediating bone remodeling, especially the CC subfamily [63]. 

**Figure 1:**
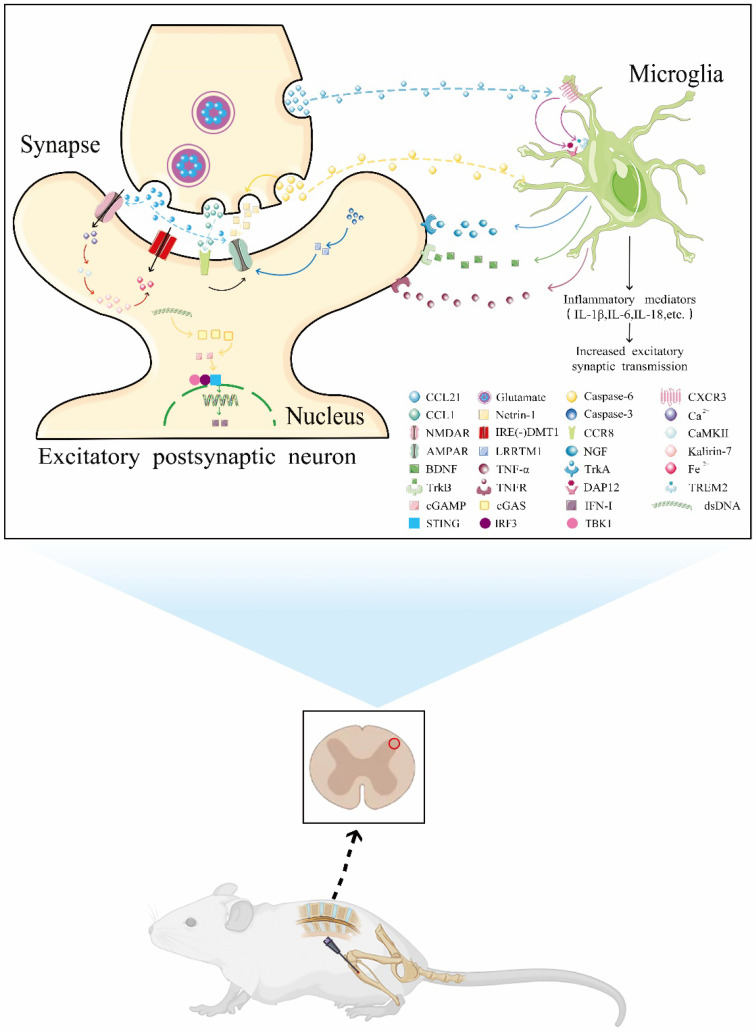
Recent insights into the molecular mechanisms of chronic pain after fractures and orthopedic surgery. Pain injury from fractures and orthopedic surgery causes mechanical deformation of somatosensory nerve endings innervating bone and muscle, and the hyperexcitability of primary sensory neurons triggers the release of multiple cytokines from the presynaptic membrane of primary nociceptive sensory neurons, which are involved in CFP processes. These mainly include: (1) CCL21 released from presynaptic neurons acts on its specific receptor CXCR3 in microglia, which promotes TREM2/DAP12 complex formation and induces microglial activation; (2) the enhanced activity of caspase-6 in the presynaptic membrane can directly act on microglia to accelerate their activation, and it can also promote the release of Netrin-1 and promote the postsynaptic transport of AMPA receptors; (3) the increased secretion of glutamate-containing vesicles located in the presynaptic membrane into the synaptic cleft can positively regulate AMPA receptors or promote the phosphorylation of NMDA receptors to increase Ca^2+^ influx, and the increased secretion of CaMKII stimulates the expression of kalirin-7, which acts on IRE(-)DMT1 to promote Fe^2+^ influx and contributes to iron overload-related neurotoxicity; (4) CCL1 released from the presynaptic membrane can directly act on the specific receptor CCR8 located in the postsynaptic membrane, thereby promoting the phosphorylation of AMPA receptors and leading to central sensitization; (5) increased activity of caspase-3 in postsynaptic membrane promotes enhanced expression of LRRTM1, which is involved in AMPA receptor postsynaptic transport and synapse formation; (6) following the activation of microglial proliferation, a series of inflammatory factors are released that can directly increase excitatory synaptic transmission; (7) dsDNA stimulates cytoplasmic cGAS to produce cGAMP, which acts on the STING-TBK1-IRF-3 axis to promote clearance of pathogens and damaged host cells by inducing nuclear IFN-Is. The above series of reactions drive the process of central sensitization through the positive regulation of NMDA receptors or AMPA receptors or directly affect the transduction of excitatory signals at the postsynaptic membrane and participate in the development of the CFP process.

CCL1, belonging to the CC subfamily of chemokines, is involved in the central regulatory process of thermal hyperalgesia and tactile allodynia by activating excitatory glutamatergic receptors in the spinal cord. CCR8 is a specific receptor for CCL1 and is mainly expressed by FOXp3+ Tregs [64]. Barsheshe Y et al. observed that in the central nervous system, CCR8+ Tregs are autocrinally enhanced by CCL1 to act as “driver” regulatory cells that inhibit disease progression [65]. Based on the above studies, Wang C et al. found [66] that orthopedic surgery-induced tibial fractures induce and maintain CFP with upregulation of CCL1/CCR8 expression and GluA1-containing AMPA receptor phosphorylation in the spine. Central CCL1/CCR8 inhibition impairs mechanical allodynia and cold hyperalgesia, presumably by inhibiting phosphorylation of GluA1-containing AMPA receptors in the dorsal horn of the spinal cord. Intrathecal injection of NASPM (a GluA1-containing AMPA receptor antagonist) attenuates CFP. These results suggest that spinal CCL1/CCR8-regulated phosphorylation of GluA1-containing AMPA receptors is involved in the regulation of mouse CFP progression (Figure 1). 

CCL21 is a microglia-activating chemokine synthesized by damaged neurons and transported through axons and involved in the regulation of pain sensation through microglia-mediated excitatory synaptic transmission [67]. Intrathecal injection of CCL21 rapidly induced pain perception in mice, while CCL21-neutralizing antibody reduced pain-like behavior in mice, and blocking its cognate CXCR3 receptor with (+/−)-NBI-74330 had a similar effect [68]. CCL21-deficient mice fail to exhibit tactile allodynia [69].

The triggering receptor expressed on myeloid cells 2 (TREM2) forms a receptor complex with DNAX-activating protein of 12 kDa (DAP12) on the plasma membrane of microglia and induces the activation of microglia via DAP12, which, in turn, is involved in the induction and maintenance of neuropathic pain [70]. Based on the studies described above, we found [71] that exposure of the spinal cord to CCL21 upregulates TREM2 and DAP12 expression, and pharmacological inhibition of TREM2/DAP12 ameliorates CFP and CCL21-induced acute pain. These results suggest that inhibition of CCL21-dependent TREM2/DAP12 neuroinflammatory signaling may have a previously undescribed role in alleviating CFP.

The above studies again strongly confirm the important role of chemokine CC in stimulating the microglia to participate in the induction and maintenance of CFP. However, in the large family of chemokines, whether there are other chemokines directly involved in the production of CFP remains unknown. At the same time, determination the intrinsic genetic mechanisms associated with the currently known chemokines involved in the process of CFP still needs further study.

#### 3.3.4. Kallikrein–Kinin System

The appearance of local tissue damage and inflammation after fracture leads to activation of the kallikrein system. Kallikrein is essentially a proteolytic enzyme that produces bradykinin (BK), a related peptide (Lys-BK), and its active metabolites from kininogen substrates [72]. Kinin is involved in the regulation of inflammatory processes by activating two G protein-coupled receptors, B1 and B2 [71]. B1R, encoded by the gene BDKRB1, is rarely expressed in healthy tissue, and its expression is only induced under specific conditions such as injury and inflammation, whereas B2R is expressed continuously [73]. In a study by Minville V et al. [74], B1R and B2R mRNA and protein levels were significantly enhanced at the fracture site. B1KO and B2KO or B1R and B2R antagonists obviously reduced CFP sensitivity in mice. Therefore, B1R and B2R antagonists appear to be potential therapeutic agents for CFP. Currently there are B1R antagonists that have been developed and are undergoing clinical testing. However, none of the B1R antagonists being tested have been approved for clinical use. Henceforth and for a long time to come, the development of B1R and B2R antagonists will be the focus of our research.

#### 3.3.5. Neurotrophins

The neurotrophins includes NGF, brain-derived neurotrophic factor (BDNF), neurotrophin 3, and neurotrophin 4. Of these, NGF and BDNF play key roles in regulating neuronal synaptic function.

One of the main function of NGF is the initiation and maintenance of hypersensitivity to pain. It may drive bone pain by inducing the sprouting of TrkA+ sensory and sympathetic nerve fibers in joints and bones [75]. Activated TrkA increases neuropeptidergic signaling, leading to the upregulation of capsaicin receptor (TRPV1) expression and BDNF, which are molecules involved in the regulation of peripheral and central hyperalgesia. Hindpaw skin expressing both mRNA and protein levels of NGF-β was significantly increased 4 weeks after fracture, supporting the hypothesis that NGF is involved in the development of CFP [76]. The application of neutralizing anti-NGF monoclonal antibodies or low-molecular-weight Trk kinase inhibitors blocks NGF/TrkA signaling, thereby reducing pain-related behaviors in rodent fracture models [77,78]. Anti-NGF administration before/after fracture or surgery reduces bone pain behavior by 31% to 70%, depending on assessed pain endpoints [79], and its analgesic effects did not appear to dissipate over time and did not impair bone repair [75]. For the above reasons, anti-NGF therapy appears to be an effective target for CFP inhibition. However, Anti-NGF therapy may have serious side effects, including peripheral edema, arthralgia, pain in the extremities, and neurosensory symptoms (e.g., paresthesia, dysesthesia, and hyperalgesia), and may even lead to the deterioration of osteoarthritis and osteonecrosis in severe cases [80]. 

BDNF is a key signaling molecule that is mainly expressed in small and medium DRG neurons and stored in large, dense core vesicles (LDCVs) [81]. It mediates neuronal survival and differentiation activities by binding and activating protomycin receptor kinase B or TrkB, a member of the larger Trk receptor family [82]. Using a tibial fracture model with intramedullary pinning [83], it was shown that pinned tibial fractures induce cold hyperalgesia and upregulate BDNF protein levels for up to 2 weeks after the intervention. This means that BDNF may be involved in the production and maintenance of CFP. After the synthesis and release of BDNF by microglia, it can increase the excitability of neurons by causing de-inhibition of neurons in the dorsal horn of the spinal cord and then participate in the production and development of CFP [84]. Of course, even though the fact that BDNF acts on the CNS to participate in the generation and maintenance of pain sensation is a well-established implementation, its specific connection with CFP is still the focus of our attention.

## 4. Commonly Used Clinical Analgesics

### 4.1. NSAIDs

Non-steroidal anti-inflammatory drugs (NSAIDs), one of the most commonly used types of clinical analgesic, mainly act on cyclooxygenase (COX) by inhibiting the synthesis of the inflammatory mediators prostaglandins (PGs), thereby reducing inflammation and achieving an analgesic effect. Cyclooxygenases are the rate-limiting enzymes in the synthesis of PGs and are divided into two categories: COX-1 and COX-2. Normally, COX-1 is widely expressed in humans, while COX-2 is usually only expressed in small amounts.

When the body experiences stress responses such as trauma and inflammation, the expression of COX-2 increases, which stimulates the synthesis and accumulation of the inflammatory mediators PGs and induces an inflammatory response. Traditional NSAIDs are potent analgesics that inhibit COX-1 and COX-2 and can shorten hospital stays when used correctly [85], but they have severe renal, gastrointestinal, and cardiotoxicity effects that limit their use in the clinical setting [86]. Selective COX-2 inhibitors that can selectively inhibit COX-2 with little effect on COX-1, resulting in a significant reduction in gastrointestinal side effects, have been discovered. However, COX-2 is required for the generation of osteoblasts during fracture healing. Inhibition of COX-2 inhibits bone healing, in particular, prolonged use of COX-2 inhibitors (>6 weeks) has serious consequences, such as delayed bone healing, nonunion, and increased risk of secondary fractures [87,88]. However, ibuprofen, which is relatively specific among NSAIDs, has gained a place in the analgesic field because of its strong analgesic effect and small effect on fracture healing [89,90,91]. At present, most researchers suggest that NSAIDs can be mainly used as pain-relief drugs in the acute phase after fracture or for short-term use (<2 weeks), and long-term use of such drugs is not recommended (Table 1).

### 4.2. Opioids

Traditional opioid analgesics include morphine, codeine, and pethidine. These drugs inhibit afferent pain impulse stimulation by reducing the release of neurotransmitters such as acetylcholine, norepinephrine, dopamine, and substance P. They are mainly used for the treatment of moderate to severe pain in clinical practice [92]. In addition to the well-known tolerance and dependence caused by long-term use, opioids have many other side effects. Recent studies have confirmed that the use of opioids in the elderly produces a variety of side effects including cognitive decline, increased sputum production, decreased blood oxygen saturation, and constipation [93]. A study by Chrastil J et al. confirmed that morphine treatment leads to weakening of calluses and thus affects fracture healing [94]. Patients using opioids are at increased risk of fracture [95,96,97], which may be due to acute central nervous system effects or the suppression of endogenous hormone production [98,99,100]. Among the many opioids available, remifentanil has a special status. Studies have shown that remifentanil can upregulate two key osteogenic transcription factors, namely runt-related transcription factor 2 (Runx2) and osterix, and promote osteoblast differentiation in vitro [101]. It also inhibits RANKL-induced osteoclast differentiation and maturation, thereby reducing bone resorption [102]. This evidence suggests that remifentanil is involved in promoting bone formation and healing. At present, opioid abuse and misuse remain major public health problems. Thus, a more stringent use system is required to effectively avoid side effects that may affect patients’ ability to lead normal lives. Although opioids are still the main analgesics used in the three-step treatment, it is currently recommended in clinical practice to minimize their use and replace them with other analgesics if the situation permits (Table 2).

### 4.3. NMDA Receptor Antagonist

The particular method by which NMDA receptors contribute to the onset and development of CFP has been covered in the preceding section. This suggests that NMDA receptor antagonists might be quite effective in treating CFP. In fact, our research has demonstrated that the specific NR2B antagonist Ro25-6981 does reduce post-fracture pain [31]. Ketamine and memantine, two examples of non-competitive NMDA receptor antagonists, have also been used for their analgesic properties [103]. Conantokins, a newly discovered selective NMDA receptor antagonist, also have a great deal of potential since they may reduce pain in a dose-dependent way without causing any significant side effects [104]. The NMDA receptor antagonists described above have significant potential for CFP treatment, not with standing the need for more study and clinical testing.

### 4.4. Anti-Absorbers

#### 4.4.1. Calcitonin

Calcitonin is an effective osteoclast receptor inhibitor, and its possible analgesic mechanism is to induce an increase in the level of endorphins, reduce the synthesis of humoral factors such as prostaglandins, or participate in central pain transmission to regulate pain sensation [105]. Karponis et al. demonstrated that calcitonin has a significant analgesic effect on distal radius fractures, and its analgesic effect starts and peaks 10 days after fracture and lasts until 45 days post-fracture [106]. Furthermore, in a meta-analysis by Knopp-Sihota et al. [107], calcitonin was shown to significantly reduce the severity of acute pain in recent osteoporotic vertebral compression fractures (VOCF) but not chronic pain severity. In addition to VOCF, calcitonin can be used as a short-term analgesic therapy for acute traumatic medullary fractures [108], mandibular fractures [109], and vertebral fractures [110]. However, intranasal calcitonin can easily cause headaches, rectal suppositories can cause gastrointestinal disorders and dizziness, and intramuscular injections can cause facial flushing, anorexia, and gastrointestinal irritation [111]. The number of analgesics available is gradually increasing, and due to its potential side effects, calcitonin has been withdrawn from the stage of first-line analgesics and is only used as a short-term drug in combination with other drugs (Table 3).

#### 4.4.2. Bisphosphonates

Bisphosphonates, generally used for the prevention of osteopenia, can be selectively used in the treatment of bone pain. Bisphosphonates can reduce the activity of osteoclasts, resulting in an analgesic effect [112]. However, several studies using animal models have shown that bisphosphonates reduce the toughness of bone tissue and negatively affect the tissue quality of aged bones [113,114]. At the same time, their analgesic effect does not directly impact the pain transmission process and is limited. Therefore, bisphosphonates are not the first choice for suppressing bone pain and are often used in combination with other drugs.

### 4.5. Vitamin D

A fat-soluble vitamin called vitamin D is known for its effects on the body’s calcium and phosphorus levels as well as bone metabolism. The human body exclusively produces vitamin D through skin synthesis, while liver and kidneys convert synthetic vitamin D into 1,25-(OH)_2_D_3_ (calcitriol) before it becomes active. According to earlier research, calcitriol can speed up the healing of fractures by controlling the Ca^2+^ equilibrium and boosting osteoblast activity. Recent research has demonstrated that vitamin D and its receptors can contribute to the pain signal transduction of opioid receptors [115], glial-derived neurotrophic factor (GDNF) [116], epidermal growth factor receptor (EGFR) [117], and other signaling pathways, therefore decreasing chronic pain. 

However, excessive vitamin D supplementation also has certain toxic effects. Taking high-dose vitamin D increases the risk of falls and fractures in postmenopausal women [118]. Hypercalcemia is one of the common toxic reactions of overdose of vitamin D, accompanied by vomiting, fatigue, anorexia, and other reactions [119]. In severe cases, vitamin D toxicity can even lead to acute kidney injury (AKI) [120]. Therefore, it is often used in combination with bisphosphonates to seek analgesic effect with high efficiency and low side effects.

## 5. Emerging Analgesic Targets or Drugs

### 5.1. STING

Stimulator of interferon genes (STING), a key sensor of DNA and an innate immune modulator [121], senses cyclic dinucleotides from bacterial sources or from cyclic GMP-AMP (2′-3′) in the cytosol and promotes the body to eliminate pathogens and damaged cells by inducing the production of type I interferon (IFN-I) [122,123]. STING agonists could rapidly suppress nociception. Wang K et al. [124] found that treatment with STING agonists (DMXAA and ADU-S100) significantly reduced mechanical allodynia and cold hyperalgesia for up to 24 h after fracture and conferred modest protection at both day 14 and day 42 post injury. Moreover, STING does not activate addiction and reward circuits in the brain as morphine and other opioids do [125], and the analgesic effects it mediates are not affected by naloxone [122]. These studies suggest that STING is a potent target molecule for inhibiting CFP, but STING agonists are still in the experimental stage. Determining how to use STING agonists safely and efficiently in clinical research and the development of prospective STING immunotherapy drugs are the top priorities for future analgesic drug development.

### 5.2. DHA and SPMs

Past studies have shown that avoiding the transformation of acute to chronic inflammation is an effective way to prevent chronic pain, which requires specialized pro-resolution mediators (SPMs) to accelerate the resolution of acute inflammation [126]. Interestingly, SPM derived from fish oil docosahexaenoic acid (DHA) can promote the resolution of acute inflammation, thereby effectively suppressing inflammation and pain, which is highly effective and safe [127]. In our previously study [128], the following findings were obtained: (1) intravenous administration of DHA (500 μg), resolvin D1 (RvD1, 500 ng), and maresin 1 (MaR1, 500 ng) at 10 min and 24 h after tibial fractures effectively delayed CFP development, and (2) IT postoperative treatment (500 ng) with neuroprotectin D1 (NPD1), MaR1, and RvD1 and RvD5 effectively reduced mechanical allodynia and cold hyperalgesia. The above studies show that fracture and the postoperative supplementation of SPM may be effective methods for preventing the generation of CFP.

### 5.3. Artesunate

Another potential analgesic drug that needs to be highlighted is artesunate. Artemisinin is a low-toxic active derivative of artemisinin (Qinghaosu) that has been used to treat malaria [129]. Emerging research shows that artesunate is effective in suppressing acute pain caused by opioids and chemical irritants [130,131]. Repeated exposure to artesunate prevents mechanical allodynia and cold hyperalgesia from fractures at 10 and 100 μg but not 1 μg, and this effect is dose-dependent [71]. More interestingly, a single intrathecal injection of 100 μg of artesunate on 14 d after orthopedic surgery relieved the established CFP. Intraperitoneal injection of artesunate can also effectively inhibit CFP, mainly at a dose of 10 or 50 mg/kg. This may be achieved through the inhibition of CCL21 by artesunate. Then, the future of artesunate as a new strategy for controlling CFP is promising.

### 5.4. Others

In addition to the currently used analgesics and potential analgesics mentioned above, many targeted molecular inhibitors are under development, such as the caspase-3 inhibitor Z-DEVD-FMK, caspase-6 inhibitor Z-VEID-FMK, and deferoxamine, etc. Their analgesic mechanisms are described above and are not repeated here. 

## 6. Conclusions

In this review, we evaluated the important chemicals that have recently been found to play a role in the emergence of CFP. Along with reviewing the potential molecular targets that could be exploited to generate extremely strong analgesic medicines, we also analyzed the primary clinical analgesic medications used to suppress CFP. However, the following issues remain with the present study on the analgesic mechanism of CFP: (1) the development of CFP is a complex process, involving a variety of cytokines in the central and peripheral regions. The involvement of peripheral sensitization in the development of CFP is still unclear because current research on CFP primarily examines central sensitization. Due to its indispensable importance, the study of peripheral mechanisms is not only a major difficulty that must be overcome but will also become another major focus of our future research. (2) Considering the gender and species differences, future study will be necessary to establish if the mechanism of CFP found in male mice can be successfully applied to female mice and even serve as a useful reference for the analgesic treatment of human CFP in mouse research. (3) The drugs commonly used in the clinical treatment of CFP are widely used in the analgesic treatment of orthopedic diseases, and their specificity is poor. However, the research on specific molecular targeted drugs that have been developed is mostly in the experimental stage, and they are not used in clinical practice because of side effects or potential safety hazards. Early development of safe and efficient clinical analgesic targeted drugs is the top priority of future research.

The treatment of CFP remains a great challenge for clinicians. Despite the fact that some of the most widely used analgesics can offer effective pain relief, their severe side effects continue to deter doctors from using them. Fortunately, new medicines are being researched, and the preliminary findings are encouraging though more thorough research is still required to give patients better pain relief. It is sincerely hoped that the content reviewed in this article can provide an effective reference for future research on analgesic drugs.

## Figures and Tables

**Table 1:** NSAIDs and its related clinical research.

NSAIDs	References	Study Type	Study Population	Evaluation and Analysis Indicators	Results
Parecoxib	Angthong C et al., 2021 [85]	Prospective double-blinded randomized placebo-controlled trial	Unstable ankle fracture *N* = 40 Mean age: 49.3 ± 18.0	Verbal numerical rating score (VNRS); verbal numerical rating percentage (VNRP)	Parecoxib (40 mg i.v.), although not providing good perioperative analgesia, may shorten the length of hospital stay
NSAIDs	Chuang PY et al., 2016 [87]	Propensity-score-matching study	Previous hip fracture *N* = 555 Mean age: >40	Kaplan–Meier survival analysis	Patients with hip fractures taking NSAIDs had an increased risk of a second hip fracture, which was significantly related to the dose of NSAIDs taken, and older adults ≥60 years of age had a higher risk of a second fracture.
COX-2-selective NSAID	Kim H et al., 2021 [88]	Propensity-score-matched study	Patients treated with NSAIDs/COX-2 drugs after fracture surgery *N* = 8693 Mean age: ≥19	Kaplan–Meier survival analysis	NSAIDs/COX-2 inhibitors for >3 weeks after orthopedic surgery for long bone fractures may lead to nonunion or delayed union.
Ibuprofen	DePeter KC et al., 2017 [89]	Retrospective study	Children with Extremity Fractures *N* = 808 Mean age: 6 months–17 years	X-rays	Perioperative or postoperative ibuprofen use in children with long bone fractures was not significantly associated with nonunion, delayed union, or bone redisplacement.
Ibuprofen	Aliskevicious M et al., 2019 [90]	Single-center, triple-blind, randomized clinical trial	Colles fracture *N* = 95 Mean age: 42–85	Daily pain score (1–10); DASH score	Ibuprofen can provide good analgesia in patients with acute fractures while reducing the dose of opioids without serious orthopedic complications.
Ibuprofen	Nuelle JAV et al., 2020 [91]	Prospective randomized parallel single-blinded study	Children long bone fracture *N* = 95 Mean age: <16 (male); <14 (female)	Visual analog scale (VAS); X-rays	Ibuprofen does not inhibit the healing of long bone fractures in children and has no significant side effects, so it is recommended for the clinical treatment of children with fractures

*N* = the total number of participants in the trial.

**Table 2:** Opioid agonists and its related clinical research.

Opioid Agonist	References	Study Type	Study Population	Evaluation and Analysis Indicators	Results
Opioids	Dagenais-Beaulé et al., 2019 [93]	Retrospective cohort study	After elective or urgent orthopedic surgery *N* = 250 Mean age: ≥65	Confusion assessment method (CAM) score; digital pain rating scale (0–10)	Compared with the old group (>80 years old) and the young group (65–79 years old), it can be observed that the opioid consumption in the former group is lower within one week after surgery, but the side effects (disorder of consciousness, renal function damage, etc.) are significantly increased.
Opioids	Leach et al., 2017 [95]	Matched case-control study	Hip fracture *N* = 44,138 Mean age: >65	Conditional logistic regression model	Initiated/continued opioid use in older adults (>65 years) increases the risk of hip fracture.
Weak opioids, buprenorophine, strong opioids	Taipale et al., 2019 [96]	MEDALZ cohort	Alzheimer’s patients *N* = 9500 Average age: 88	Cox proportional hazard models	Long-term (>180 days) opioid use in patients with Alzheimer’s disease increases the risk of hip fracture, and this phenomenon is positively associated with opioid intensity.
Opioids	Schwarzer et al., 2018 [97]	Case-controlled study	Low-energy fractures *N* = 992 Mean age: >60	Follow-up	Adverse events such as fatigue, gastrointestinal reactions, etc., are more common in patients with low-energy fractures taking opioids.
Opioids	Li L et al., 2013 [98]	Randomized risk-set sampling trial	Noncancer patients *N* = 1,700,000 Mean age: 18–80	Conditional logistic regression model	Opioid use increases the risk of fractures in adults with noncancer pain, especially in the first few weeks of use, which is largely influenced by the acute central nervous system effects of opioids.
Opioids	Tolppanen AM et al., 2016 [99]	Exposure-matched cohort	Alzheimer’s patients *N* = 67,072 Mean age: 34–105	Cox regression	Among non-AD patients in the control group, opioid use was strongly associated with higher fracture rates.
Opioids	Acurcio FA et al., 2016 [100]	Retrospective nested case-control study	rheumatoid arthritis (RA) *N* = 9769 Mean age: >20	Follow-up	In RA patients, opioid use is associated with an increased incidence of nonvertebral fractures.

*N* = the total number of participants in the trial.

**Table 3:** Calcitonin and its related clinical research.

Calcitonin	References	Study Type	Study Population	Evaluation and Analysis Indicators	Results
Elcatonin vs. risedronate	Fujita T et al., 2011 [105]	Prospective randomized double-blind study	Chief complaint of back and/or knee pain *N* = 40 Mean age: >50	Visual rating system (VRS) (0–100); electroalgometry (EAM)	Intramuscular injection of calcitonin showed good analgesic effect in both subjective (VRS) and objective (EAM) evaluation indicators.
Nasal salmon calcitonin	Karponis A et al., 2015 [106]	Prospective randomized double-blind study	Distal radius fracture *N* = 41 Mean age: >50	Visual analogue scale (VAS) (0–10); follow-up	In patients with distal radius fractures, significant analgesic effects were seen 10 days after orthopedic surgery and persisted until 45 days after surgery.
Calcitonin	Knopp-Sihota JA et al., 2012 [107]	Systematic review and meta-analysis	recent and remote osteoporotic vertebral compression fractures (OVCF) *N* = 589 Mean age: >50	Visual analogue scale (VAS)	The efficacy of calcitonin in the treatment of acute OVCF in elderly (>60 years old) patients is worthy of recognition, and its side effects are mild (mainly gastrointestinal reactions).
Salmon calcitonin nasal spray	Roy A et al., 2021 [109]	Randomized controlled trial	Mandibular fractures *N* = 14 Mean age: 20–40	Visual analogue scale (VAS); Biochemical assessment; Radiological assessment (OPG)	Intranasal salmon calcitonin spray can inhibit postoperative pain after open reduction and internal fixation of fractures, increase plasma osteocalcin levels, and promote fracture healing.
Calcitonin nasal spray	Sun LM et al., 2014 [111]	Population-based nested case-control study	Osteoporosis *N* = 5652	Follow-up	Women with osteoporosis have an increased risk of liver cancer after CNS use.

*N* = the total number of participants in the trial.

## Data Availability

All data relevant to the research are included in the paper for figures. Data are available from the corresponding author upon reasonable request.

## Abbreviations

CFP, chronic fracture pain; CRPS, complex regional pain syndrome; AMPA, alpha-amino-3-hydroxy-5-methyl-4-isoxazolepropionic acid; CNS, central nervous system; LRRTM1, leucine-rich repeat transmembrane protein 1; CGRP, calcitonin-gene-related peptide; NMDA, N-methyl-D-aspartate; DMT1, divalent metal transporter 1; UTR, untranslated region; IRE, iron-responsive element; CGRP, calcitonin-gene-related peptide; NF-κB, nuclear factor kappa B; IL, interleukin; IL-1β, interleukin-1beta; TNF-α, tumor necrosis factor-α; RIPK3, receptor-interacting protein kinase 3; NLRP3, NOD-like receptor protein 3; BSF-2, B-cell stimulatory factor 2; CCL1, C-C motif Chemokine ligand 1; CCR8, C-C Motif Chemokine Receptor 8; CCL21, C-C Motif Chemokine Ligand 21; CXCR3, C-X-C Motif Chemokine Receptor 3; TREM2, triggering receptor expressed on myeloid cells 2; DAP12, DNAX-activating protein of 12 kDa; BK, bradykinin; Lys-BK, bradykinin related peptides; DRG, dorsal root ganglion; NGF-β, nerve growth factor β; BDNF, brain-derived neurotrophic factor; GDNF, glial-derived neurotrophic factor; EGFR, epidermal growth factor receptor; TRPV1, transient receptor potential vanilloid-1; LDCVs, large dense core vesicles; NSAIDs, non-steroidal anti-inflammatory drugs; COX, cyclooxygenase; PGs, prostaglandins; Runx2, runt-related transcription factor 2; VOCF, osteoporotic vertebral compression fractures; STING, stimulator of interferon genes; IFN-I, type I interferons; SPMs, specialized pro-resolving mediators; DHA, docosahexaenoic acid; RvD1, resolvin D1; MaR1, maresin 1; NPD1, neuroprotectin D1; RvD5, resolvin D5.
